# Titanium Phosphate Nanoplates Modified With AgBr@Ag Nanoparticles: A Novel Heterostructured Photocatalyst With Significantly Enhanced Visible Light Responsive Activity

**DOI:** 10.3389/fchem.2018.00489

**Published:** 2018-10-17

**Authors:** Manli Ren, Jiaqiu Bao, Peifang Wang, Chao Wang, Yanhui Ao

**Affiliations:** Key Laboratory of Integrated Regulation and Resource Development on Shallow Lakes, Ministry of Education, College of Environment, Hohai University, Nanjing, China

**Keywords:** heterojunction, titanium phosphate, AgBr@Ag, photocatalysis, visible light

## Abstract

AgBr@Ag modified titanium phosphate composites were fabricated through a two-step approach. The prepared samples were characterized by X-ray diffraction, scanning electron microscopy, and transmission electron microscopy. The optical properties of the composites were characterized by using UV-vis diffuse reflectance spectroscopy. The photocatalytic activities of the composites were investigated on the degradation of Rhodamine B and ciprofloxacin under visible light irradiation. AgBr@Ag/titanium phosphate was determined to exhibit considerably higher photocatalytic activity than the corresponding individual components. The mechanism on the enhancement of the photocatalytic activity was proposed based on the results of photoluminescence spectra and photocurrent measurements. Furthermore, the possible photocatalytic mechanisms of organic compounds degradation were also proposed.

## Introduction

Environmental pollution has become a global problem due to the accelerated development of human society (Ye et al., 2012; Sun et al., 2017; Zhang et al., 2017; Li et al., 2018a; Lu et al., 2018). Therefore, it is urgent to solve this severe problem via green technology (Liu et al., 2012; Hao et al., 2016; Zou et al., 2016; Chen et al., 2018; Feng et al., 2018; Guo et al., 2018). Currently, photocatalysis technology has recieved intense attention as an efficient technology for solving the problem of environment (Fan et al., 2013; Ao et al., 2016; Wang et al., 2016; Dong et al., 2017; Hao et al., 2017). Furthermore, it is well known that composite photocatalysts always show higher activity than the corresponding single component (Ma et al., 2018; Wang et al., 2018; Zhang et al., 2018). The improved photocatalytic activity is due to the different electronic energy levels of different components, which create an internal electric field that can accelerate the separation rate of photogenerated electrons and holes, and reduce their recombination rate (Guo et al., 2017).

Recently, titanium phosphate has been widely studied for the treatment of organic compounds. Titanium phosphate [α-Ti(HPO_4_)_2_•H_2_O, hereafter TP] consists of layers of titanium atoms bonding on both plane sides of the monohydrogen phosphate groups. Water molecules located in the interlayer region to form a network of hydrogen-bonding with phosphate groups (Ekambaram et al., 2000; Guo et al., 2013; Guo and Han, 2014; Zhu et al., 2014). However, titanium phosphate can only absorb ultraviolet light (<5% of the sunlight). Furthermore, the high recombination rate of photoinduced electron-hole pairs is still exist, which would lead to lower photocatalytic activity. Thus, it is urgent to find effective ways to improve the activity of the TP.

Recently, silver halide materials have received intense attention due to their excellent visible light responsive photocatalytic activity. Silver halide@silver nanoparticles (AgNPs) based heterojunctions have been found to be excellent photocatalysts (Chen et al., 2015; Bai et al., 2016; Li et al., 2018b; Xuan et al., 2018). It has been proved that plasmon AgNPs on the surface of AgX can enhance the light absorption of the composites and accelerate the separation rate of the photogenerated charges (Daupor and Wongnawa, 2014; Li et al., 2015a,b; Ding et al., 2018; Xiao et al., 2018). Moreover, it was also considered that AgNPs decrease the recombination rate of Ag^+^ with photogenerated electrons, and allow the formation of Br^0^ species (photogenerated holes react with Br^−^ ions) that can degrade the organic compounds (Xia and Halas, 2005; Jiang et al., 2014; Zhang et al., 2015a). Therefore, it would be a good way to obtain higher visible light activated activity through the conbination of AgBr@Ag and TP.

In this work, we prepared visible light active AgBr@Ag/TP photocatalysts by a simple and feasible method. The morphology, crystal structure, optical properties, and composition of the photocatalysts were investigated. Moreover, the possible photocatalytic degradation mechanism of ciprofloxacin and the enhancement mechanism photocatalytic activity were proposed.

## Experimental

All experimental details are shown in Supporting Information.

## Results and discussion

### Characterization

**Figure 1:**
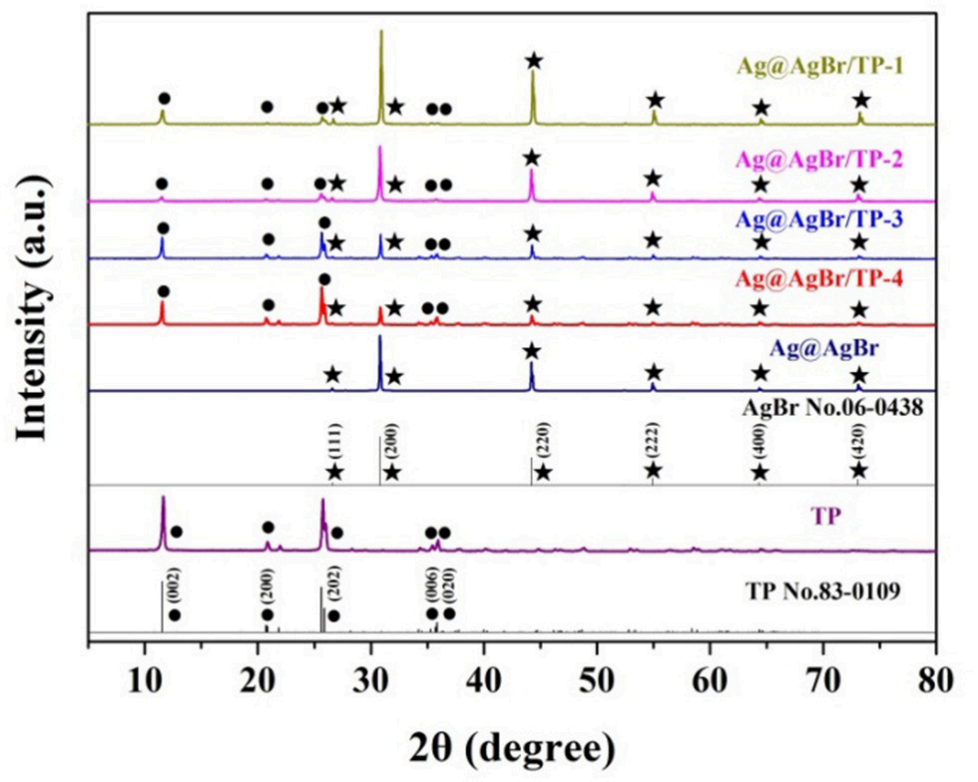
XRD patterns of pure titanium phosphate, Ag@AgBr and Ag@AgBr/TP. The star represents AgBr.

The phase structure was characterized by XRD, and the obtained patterns are shown in Figure 1. The peaks at about 11.6, 21.0, 26.0, 35.4, and 35.8° are indexed to the (002), (200), (202), (006), and (020) directions, respectively. All peaks can be ascribed to TP (JCPDS Card No: 83-0109; Ortíz-Oliveros et al., 2014; Yada et al., 2014). Diffraction peaks are also detected at 26.73, 30.96, 44.35, 55.04, 64.49, and 73.26°, which can be indexed to the (111), (200), (220), (222), (400), and (420) directions of AgBr (JCPDS Card NO: 06-0438; Chen et al., 2015; Bai et al., 2016). Furthermore, with the increasing of AgBr mass, the intensity of the characteristic peaks of AgBr increased gradually. No diffraction peaks of Ag can be observed, possibly due to its low mass, high dispersion or small size (Wang et al., 2013, 2014).

**Figure 2:**
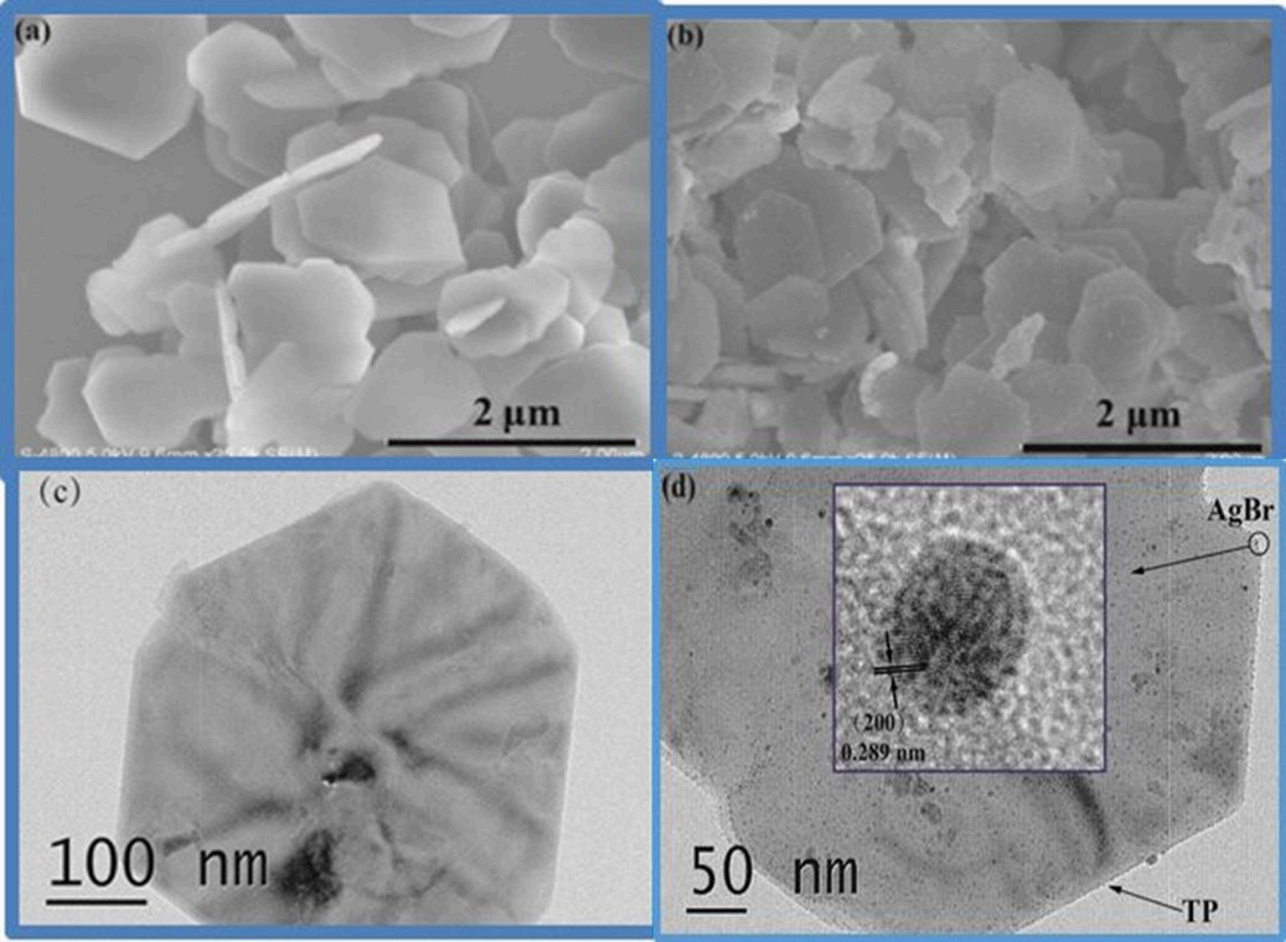
**(a)** SEM image of pure titanium phosphate, **(b)** SEM image of Ag@AgBr/TP-3, **(c)** HRTEM image of pure titanium phosphate, **(d)** HRTEM image of Ag@AgBr/TP-3.

The morphologies of the composites were investigated by TEM and SEM. The obtained SEM image of pure TP is displayed in Figure 2a. It can be seen that the TP sample is composed of uniform plates with sub-micrometer width and 30~40 nm thickness. The SEM image of AgBr@Ag/TP-3 taken from Figure 2b displays smaller AgBr@Ag nanoparticles decorating the TP surface. The TEM image of pure TP is displayed in Figure 2c. It can also be seen that the titanium phosphate plate is hexagonal, which is in good agreement with the results of SEM. The TEM image of AgBr@Ag/TP is shown in Figure 2d. It can be seen that AgBr nanoparticles decorated the TP surface, indicating the formation of heterojunctions in the prepared samples (Cui et al., 2018). As shown in the inset of Figure 2d, the measured lattice spacings of 0.289 nm corresponds to the (200) lattice planes of AgBr.

**Figure 3:**
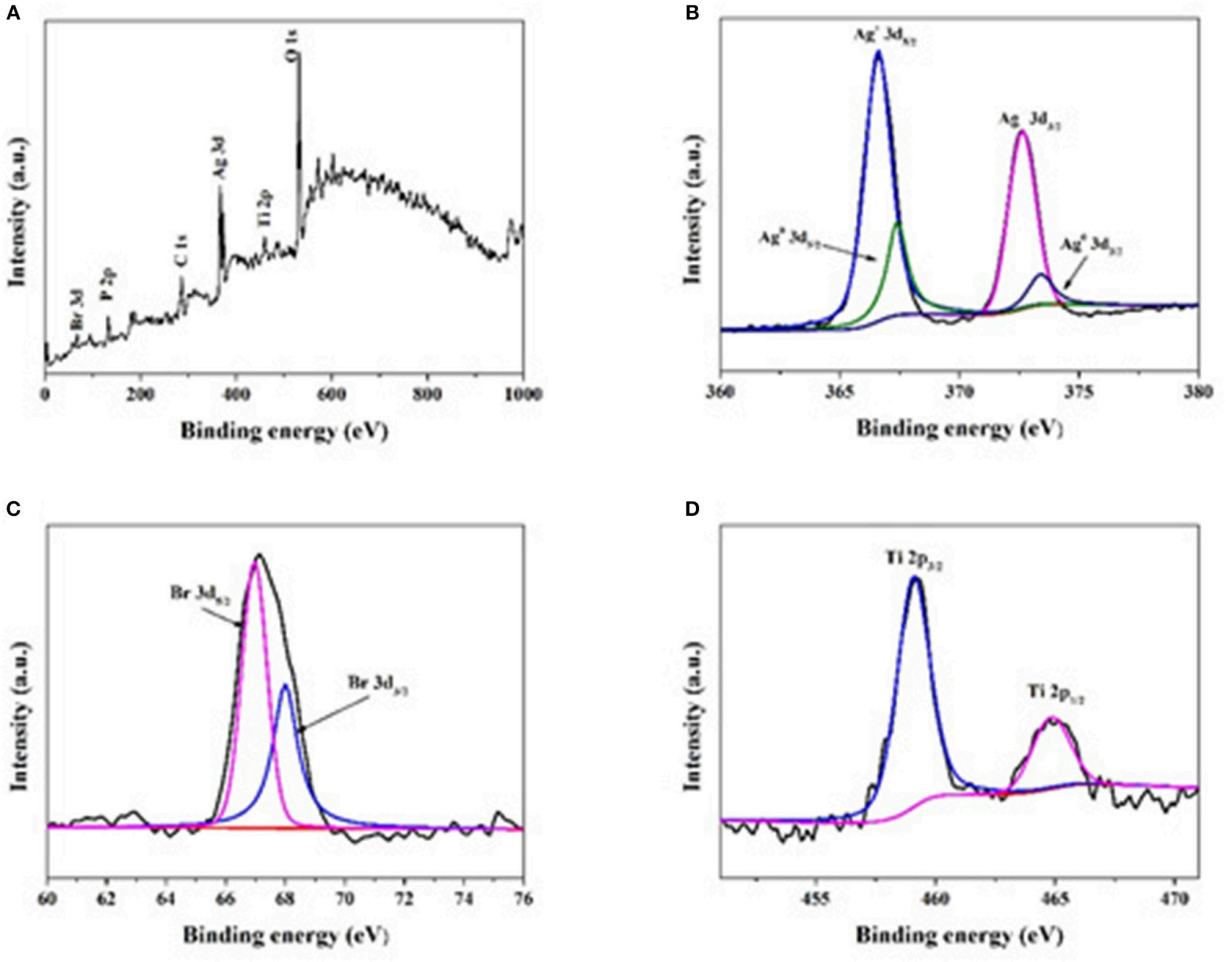
XPS spectra of Ag@AgBr/TP-3. **(A)** The survey XPS spectrum, **(B)** Ag 3d, **(C)** Br 3d, **(D)** Ti 2p peaks related to the photocatalyst.

The elemental composition and chemical states of the elements in the composites were analyzed by XPS. The peaks of Br 3d, P 2p, Ag 3d, C 1s, Ti 2p, and O1s can be observed in Figures 3A,B shows the electron binding energies of Ag 3d_5/2_ and Ag 3d_3/2_ orbitals, which can be further divided into 366.6/367.4 and 372.6/373.4 eV, respectively. The peaks at 366.6 and 372.6 eV indicate the presence of Ag^+^. Both the peak separation (6.0 eV) and peak positions (367.4 and 373.4 eV) indicate the presence of metallic form (Ag^0^; Yang et al., 2015; Tang et al., 2017a,b). As shown in Figure 3C, two peaks at 68.0 and 67.0 eV corresponding to the binding energies of Br 3d_3/2_ and Br 3d_5/2_ can be found (Wang et al., 2009). All above results prove the presence of AgBr and Ag in the composite. In Figure 3D, the Ti peaks observed at 459.0 and 464.8 eV can be attributed to Ti 2p_3/2_ and Ti 2p_1/2_. The peak separation of 5.8 eV for binding energy between the two peaks indicates that Ti was mainly Ti^4+^ in the composite (Yada et al., 2014).

**Figure 4:**
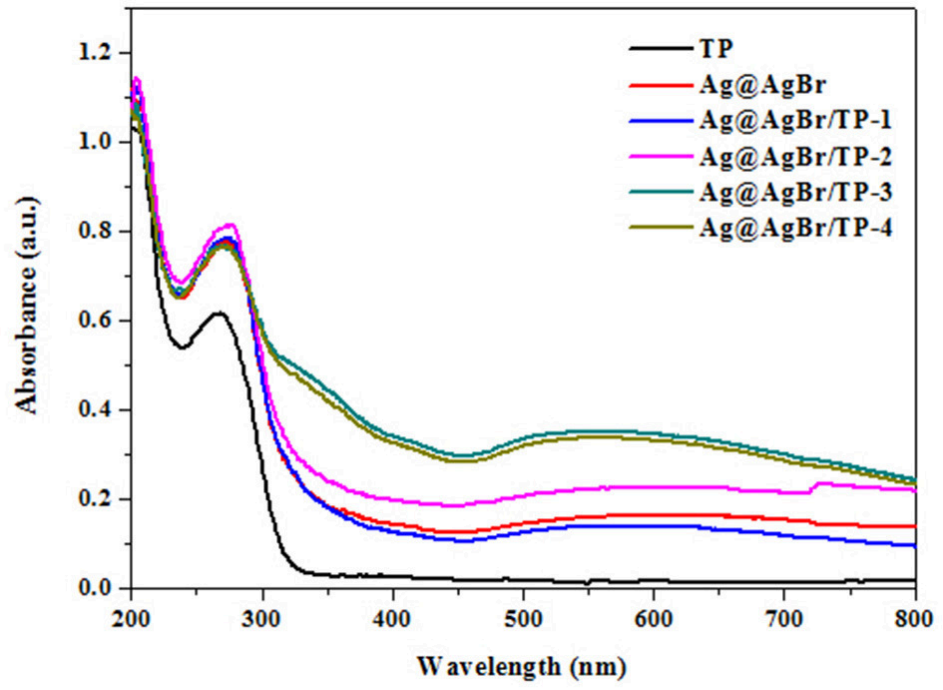
UV-vis diffuse reflectance spectra of titanium phosphate, Ag@AgBr and Ag@AgBr/TP.

The optical properties of photocatalysts significantly affect their photocatalytic performance (He et al., 2015). Therefore, optical properties of the composites were investigated by DRS and the obtained results are shown in Figure 4. Pure TP exhibited no absorption in the visible-light region, whereas the Ag@AgBr/TP composites had better absorption performance, possibly due to the deposition of AgBr and formation of AgNPs on the surface of the composites. Moreover, Ag@AgBr/TP-3 showed the highest absorption in visible light region, indicating that high absorption capability is ascribed to an optimal loading. In other words, when the loading of AgBr@Ag exceeds a critical value, the AgBr@Ag nanoparticles would agglomerate and decrease the absorption capability.

### Photocatalytic activity

**Figure 5:**
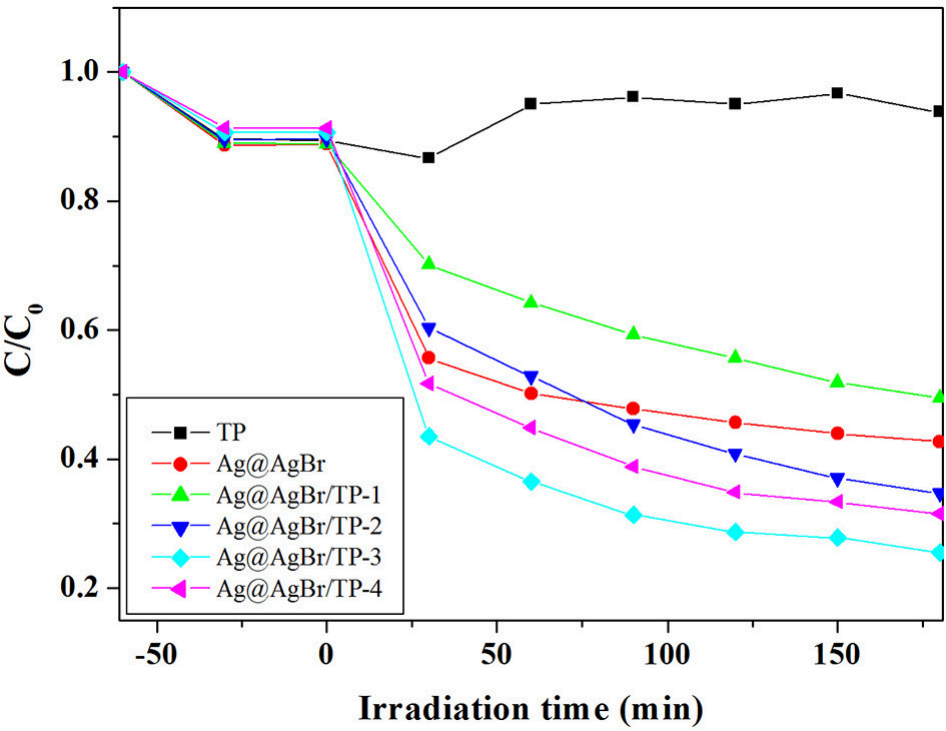
Photocatalytic degradation of CIP over the as-prepared samples under visible light irradiation.

To investigate the photocatalytic performance of the composites, we conducted experiments on the degradation of colorless organic pollutant (CIP) under visible light irradiation. As shown in Figure 5, AgBr@Ag/TP-3 displayed the highest photocatalytic activity for CIP degradation among all samples. Approximately 51.4, 44.2, 61.3, 71.5, and 65.4% of CIP was degraded in 180 min by AgBr@Ag, AgBr@Ag/TP-1, AgBr@Ag/TP-2, AgBr@Ag/TP-3, and AgBr@Ag/TP-4, respectively. Furthermore, the TP showed a low photocatalytic efficiency for CIP degradation. The degradation curve of CIP by TP exhibits a fluctuating trend. There may be some CIP molecules desorbed from the surface of TP. These results show that the Ag@AgBr/TP photocatalysts are generally effective for the degradation of colorless organic compounds.

### Intermediates analysis

**Figure 6:**
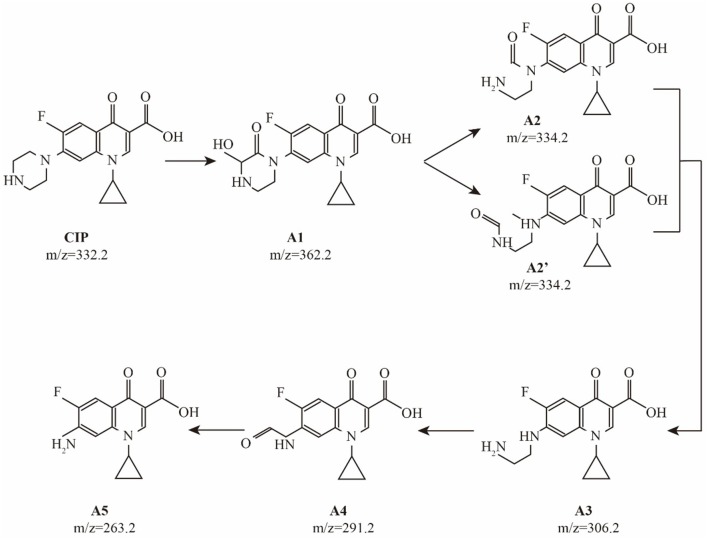
Suggested pathways for CIP degradation.

Prior to the complete mineralization of CIP, there are many degradation intermediates in the photocatalytic process. The sample solution was detected by HPLC/MS during the photocatalytic degradation. Some intermediates in the photocatalytic process were identified: A1 (m/z = 362.2), A2 (m/z = 334.2), A3 (m/z = 306.2), A4 (m/z = 291.2), and A5 (m/z = 263.2). These results show that A1~A5 are the main intermediates during the degradation process of CIP. As observed in many other oxidation system (Sturini et al., 2012), desethylene ciprofloxacin (A3) was dominant intermediate of CIP, and was formed by a net loss of C_2_H_2_ at the piperazinyl substituent of CIP. Prior to the formation of desethylene ciprofloxacin, two intermediates were detected. The hydroxyl keto-derivative of the piperazinyl substituent (A1) was detected to be the first oxidized intermediate on the way to the formation of A3. Apparently, the subsequent loss of CO from A1 results in the cleavage of the piperazinyl ring to form keto-derivatives A2 and A2', in which a carbonyl group remains on the N atom of aniline and alkylamine, respectively. A second loss of a CO molecule from A2 and A2' leads to the formation of desethylene ciprofloxacin (A3). The further oxidation of desethylene ciprofloxacin (A3) generated A4 with a keto group through the loss of a N atom. The decarbonylation of A4 forms aniline (A5), completely destroying the piperazinyl substituent of CIP. The probable pathways for CIP degradation are shown in Figure 6.

### Possible photocatalytic mechanism

**Figure 7:**
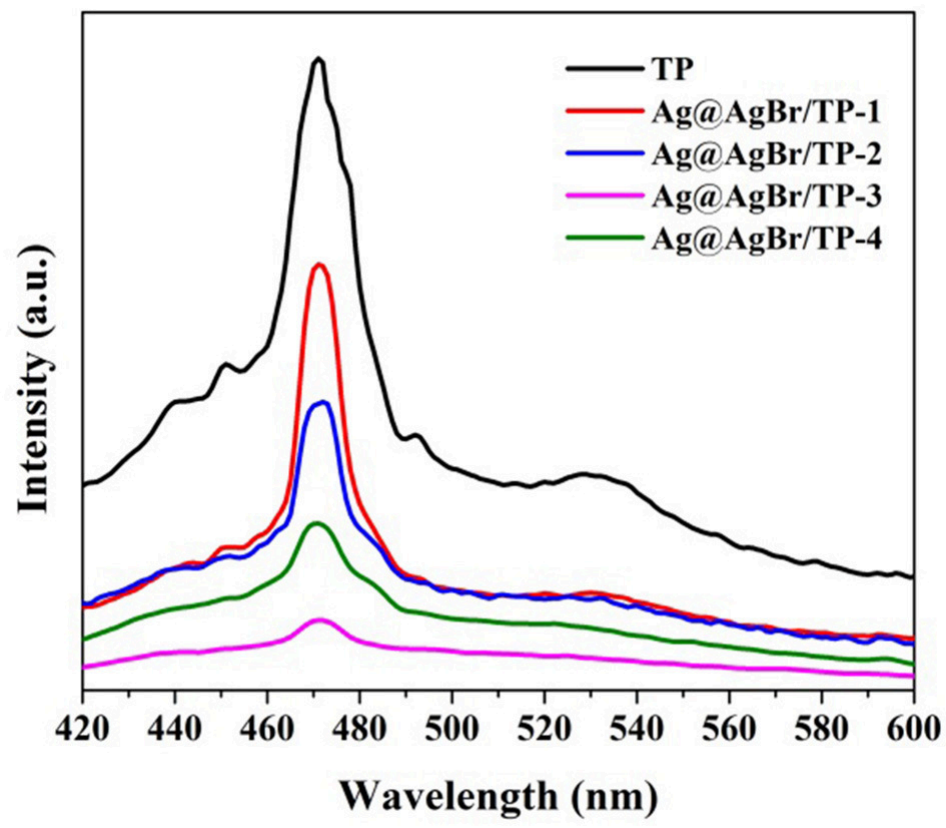
Photoluminescence spectra of the prepared samples.

Several experiments were performed to further understand the mechanism on the enhancement of photocatalytic performance for the AgBr@Ag/TP composites. The photoluminescence (PL) spectrum was measured to investigate the recombination rate of the photogenerated electron-hole pairs. The photoluminescence spectra of the prepared samples are shown in Figure 7. The intensity of PL is directly proportional to the electron-hole recombination rate, and lower PL intensity means lower recombination rate (Zhang et al., 2015b; Shi et al., 2016). The main emission peak of pure TP centers at ~470 nm, which was ascribed to the band gap recombination of electron-hole pairs. The PL intensity decreases significantly after the deposition of AgBr@Ag. The decreasing emission intensity of the AgBr@Ag/TP suggests that the recombination of electron-hole pairs can be suppressed after the deposition of AgBr@Ag (Xu et al., 2013). Furthermore, it can be seen that the PL intensity increase as the amount of Ag@AgBr increase to a certain value, and AgBr@Ag/TP-3 composite showed the lowest PL intensity, which means Ag@AgBr/TP-3 composite has the lowest recombination rate of photogenerated electrons-hole pairs. Therefore, it would lead to the highest photocatalytic performance. However, the PL intensity increase as the amount of Ag@AgBr increase further. It can be ascribed to the reason that superfluous Ag@AgBr nanoparticles would aggregate thus cannot form effective heterojunctions with TP. The above results demonstrate that the deposition of AgBr on TP and the formation of AgNPs on the surface of the composite can decrease the recombination efficiency and enhance the separation efficiency of the photogenerated electrons and holes.

**Figure 8:**
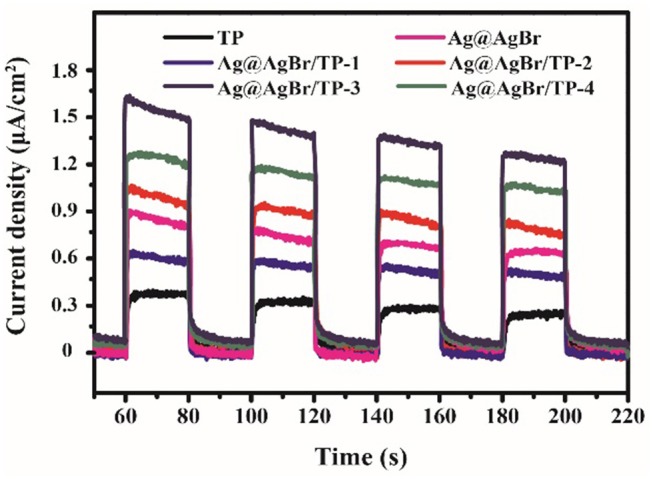
Photocurrent of the prepared samples under visible light irradiation.

It is well known that the efficiency of the electron-hole pairs separation significantly affects the photocatalytic performance (Jiang et al., 2011). The electrons in the valence band of photocatalysts can be activated and then transfer to the conduction band, leading to the formation of photocurrent. A higher photocurrent means higher electron-hole pairs separation rate, which induces to better photocatalytic performance (Xiang et al., 2011). Figure 8 shows the photocurrents for different samples under 20 s intermittent visible-light irradiation with a bias of 0.3 V. The photocurrents of AgBr@Ag/TP composites are all much higher than that of TP. Moreover, AgBr@Ag/TP-3 exhibits the highest value. The results are in good agreement with the photocatalytic activity, indicating that the efficient separation of photo-induced electrons and holes results in the high photocatalytic activity.

It was demonstrated that the separation efficiency of photo-generated electrons and holes is important to the photocatalytic performance. Furthermore, the conduction band (CB) and valence band (VB) potential have a significantly effect on the separation of the photogenerated electron-hole pairs. The CB and VB potential of AgBr and TP can be obtained using Equations. (1) and (2) (Zhang et al., 2008):

(1) $$\begin{array}{l} {\text{E}}_{\text{vb}}=\text{X}-{\text{E}}_{0}+0.5{\text{E}}_{\text{g}} \end{array}$$

(2) $$\begin{array}{l} {\text{E}}_{\text{cb}}={\text{E}}_{\text{vb}}-{\text{E}}_{\text{g}} \end{array}$$

**w**here E_vb_ is the potential of VB, X is the absolute electronegativity of photocatalyst (X is 6.85 eV and 5.81 eV of TP and AgBr, respectively), E_g_ is the band gap energy and E_0_ is the energy of free electrons (~4.5 eV). Based on these equations, the CB potential (E_cb_) and VB potential (E_vb_) of TP are calculated as 0.43 and 4.28 eV, while E_cb_ and E_vb_ of AgBr are calculated as 0.01 eV and 2.62 eV, respectively.

**Figure 9:**
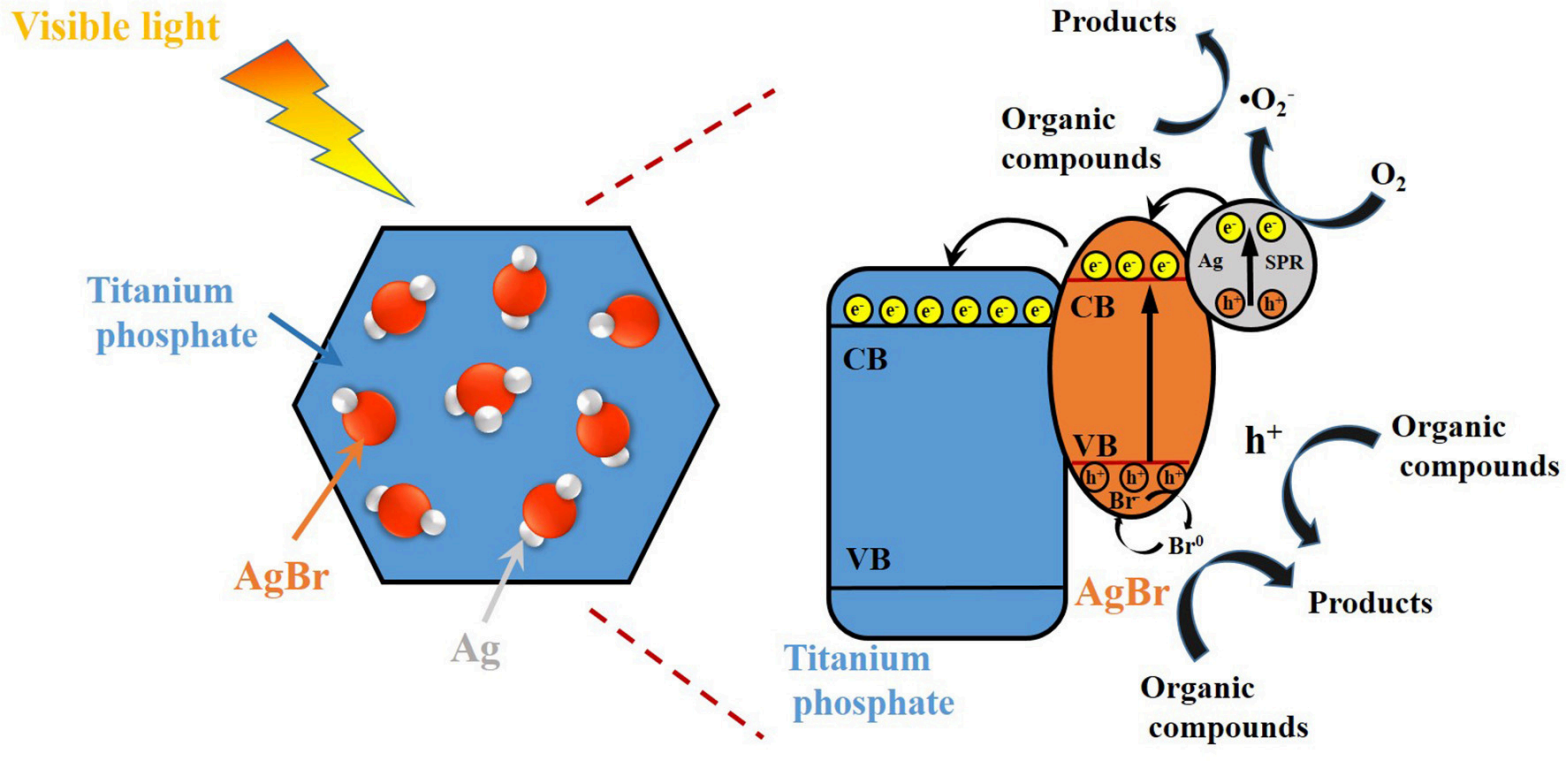
Schematic diagram of the possible photocatalytic degradation mechanism over Ag@AgBr/TP under visible light irradiation.

Therefore, a possible mechanism on the photocatalytic degradation of RhB by AgBr@Ag/TP under the irradiation of visible light is illustrated in Figure 9. The plasmon induced holes can react with Br^−^ and form active Br^0^ atoms. The Br^0^ atoms exhibit an excellent oxidation capability to oxidize organic compounds. Meanwhile, plasmon induced electrons are formed on the surface of AgNPs, part of these electrons are trapped by O_2_ to produce •${\text{O}}_{2}^{-}$, mainly contributing to the organic compounds removal (Ye et al., 2012; Zhang and Yates, 2012; Cheng et al., 2015; Ding et al., 2018). Besides, the other electrons would transfer to the conduction band of AgBr, and then further transfer from AgBr to the conduction band of TP. Meanwhile, the h^+^ stored in valence band of AgBr and TP was also involved in the organic compounds photocatalytic degradation, though they are not the dominant active species. The relevant equations are shown as following:

(3) $$\begin{array}{l} \text{Ag}+\text{visible}-\text{light}\to {\text{e}}^{-}+{\text{h}}^{+} \end{array}$$

(4) $$\begin{array}{l} {\text{e}}^{-}+{\text{O}}_{2}\to •{\text{O}}_{2}^{-} \end{array}$$

(5) $$\begin{array}{l} {\text{h}}^{+}+\text{B}{\text{r}}^{-}\to \text{B}{\text{r}}^{0} \end{array}$$

(6) $$\begin{array}{l} \text{CIP}+•{\text{O}}_{2}^{-}\left(\text{orB}{\text{r}}^{0}\right)\to \text{degradation products} \end{array}$$

Based on above results, the possible mechanism on the enhancement of photocatalytic activity for AgBr@Ag/TP composites can be proposed. AgBr@Ag nanoparticles deposited on TP improve the light absorption capability, as confirmed by UV-vis DRS studies. Furthermore, the heterojunctions between AgNPs, AgBr, and TP are conducive to improving the transfer rate and separation efficiency of the electron-hole pairs, and hindering the recombination of the pairs, thus inducing to the enhancement of the photocatalytic activity.

## Conclusions

AgBr@Ag/TP composites exhibited higher photocatalytic performance than pure TP and AgBr@Ag, and can degrade **a typical antibiotic (CIP)** under the irradiation of visible light. The excellent photocatalytic activity can be ascribed to their enhanced visible-light absorption, low recombination and high transferring rate of the photo-generated charges, as shown by DRS, PL, and photocurrent experiments.

## Author contributions

YA and CW designed the project and guided the work. MR and JB conducted the experiments and characterizations of the samples. PW revised and polished the manuscript.

### Conflict of interest statement

The authors declare that the research was conducted in the absence of any commercial or financial relationships that could be construed as a potential conflict of interest.
