# Supplementary material for: Titanium Phosphate Nanoplates Modified With AgBr@Ag Nanoparticles: A Novel Heterostructured Photocatalyst With Significantly Enhanced Visible Light Responsive Activity
Source: Front Chem. 2018 Oct 17;6:489. doi: 10.3389/fchem.2018.00489 (PMC6199560; doi:10.3389/fchem.2018.00489)
Supplement: Supplementary file 1 [file Table_1.DOCX]

**Supporting Information**

**Titanium phosphate nanoplates modified with AgBr@Ag nanoparticles: a novel heterostructured photocatalyst with significantly enhanced visible light responsive activity**

**Manli Ren, Jiaqiu Bao, Peifang Wang, Chao Wang, Yanhui Ao^[[1]](#footnote-1)^***

Key Laboratory of Integrated Regulation and Resource Development on Shallow Lakes, Ministry of Education, College of Environment, Hohai University, Nanjing, 210098, China

1. **Experimental section**
   1. **Synthesis of AgBr@Ag/TP**

The reagents are all analytic grade and are used without any purification. TP was prepared by a simple hydrothermal method as reported. Generally, 4 mL tetrabutyltitanate and 4 mL acetic acid were added into 20 mL absolute ethanol, and stirred to form solution A. 12 mL of 0.01 M hydrochloric acid, 0.04 mL ammonium hydroxide and 5.66 mL phosphoric acid were added into 20 mL absolute ethanol (defined as solution B). Then, solution B was added dropwise into solution A under stirring. The system was kept stirring for 6 h. Afterwards, the mixtures were transferred into a 100 mL of Teflon-lined autoclave. The autoclave was transferred to a oven and treated at 180 ℃ for 12 h. The obtained products were washed with ethanol and deionized water. At last, the samples were dried at 60 ℃.

The AgBr@Ag/TP hybrids was synthesized as following. 0.3 g of TP was added in 50 mL ultrapure water and ultrasound treated for 5 min. Then, AgNO_3_ solution with a certain concentration was added gradually. The solution was stirred for 1 h (wrapped with aluminum foil). Subsequently, NaBr solution with certain concentration was added dropwire and stirred for 15 min. Then, the mixtures were irradiated by visible light for 15 min. A 230 W metal halide lamp (MVL-210) was used as the visible light source (λ > 400 nm). After irradiation, the obtained samples were washed with ethanol and deionized water. At last, the samples were dried at 60 ℃. Four samples with different AgBr@Ag/TP mass ratios (20%, 100%, 150%, 200%) were obtained, and are labeled as AgBr@Ag/TP-1, AgBr@Ag/TP-2, AgBr@Ag/TP-3, AgBr@Ag/TP-4.

- 1. **Characterization**

The crystal structure of the composites were probed by X-ray diffraction (XRD, Rigaku, Smartlab). The morphology of the samples were examined by transmission electron microscopy (TEM, JEOL, JEM-2100) and scanning electron microscopy (SEM, Hitachi, s-4800). The surface elemental compositions was examined by X-ray photoelectron spectroscopy (XPS, Thermo ESCALAB 250Xi). UV-vis diffuse reflectance spectra were obained by a UV-vis spectrophotometer (Shimadzu, UV3600). Photoluminescence (PL) spectra were obtained by using a fluorescence spectrometer (Edinburgh, FLs980), **the excitation wavelength was 380 nm**.

- 1. **Photocatalytic activity**

The photocatalytic activity of the composites was probed by degradation of three organic compounds (X-3B, RhB and CIP) in aqueous solution under visible light irradiation. The visible light was provided by a 300 W Xe lamp (Zhongjiaojin yuan, CEL-HXF300) with a UV Cut-off filter that removed the light of λ < 400 nm (UVCUT400) and the luminous flux was approximately 5000 lm. Twenty mg samples was added into the RhB, X-3B and CIP (50 mL, 5 mg/L) solutions. The suspension was sonicated for 2 min and then stirred under dark for 1 h to achieve adsorption-desorption equilibrium befor irradiation,. After irradiation, approximately 1.5 mL suspension was sampled at certain time intervals for measuring the concentration of the organics.

- 1. **Photoelectrochemical experiment**

The working electrodes were samples coated on FTO with an effective area of 1.0 × 1.0 cm^2^, which were prepared by a drop-casting method. 10 mg samples were added into 0.2 mL ethanol, and sonicated for 1 h to form a uniform slurry. The slurry was spread onto the conductive surface of FTO to form a uniform film. Then the obtained electrodes were dried at 200 ℃ for 1 h. The photocurrents were examined using an electrochemical station (Chenhua Instruments, CHI660D). A saturated calomel electrode (SCE) was used as reference electrode and a Pt foil as counter electrode, while the above prepared films were working electrodes. A 230W metal halide lamp (MVL-210) was used as a visible light source (λ > 400 nm). Na_2_SO_4_ solution (0.1 M) was adopted as the electrolyte.

- 1. **Analysis**

The intermediates of CIP in the solution during the photocatalytic process was determined by the HPLC/MS method. The mobile phase was composed of water (A) and acetonitrile (B) containing 0.1% formic acid (v/v) and the flow rate was 0.4 mL/min. The gradient was started with 20% B, increased to 30% within 2.5 min and to 80% within 3.5 min, and later returned to the initial composition within 5 min and equilibrated within 4 min. The injection volume was 20 μL, and the column temperature was 30 ℃.

1. * Corresponding author. Tel./ fax: +86 25 83787330,

   E-mail address: [andyao@hhu.edu.cn](mailto:andyao@hhu.edu.cn) (Y.H. Ao) [↑](#footnote-ref-1)
